# Use of Endovascular Simulator in Training of Neurosurgery Residents – A Review and Single Institution Experience

**DOI:** 10.7759/cureus.11931

**Published:** 2020-12-06

**Authors:** Tye Patchana, James Wiginton, Hammad Ghanchi, Andrew W Favre, Emilio C Tayag, Michael Schiraldi, Dan E Miulli

**Affiliations:** 1 Neurosurgery, Riverside University Health System Medical Center, Moreno Valley, USA; 2 Medicine, Lake Erie College of Osteopathic Medicine, Bradenton, USA; 3 Neurology and Neurosurgery, Desert Regional Medical Center, Palm Springs, USA; 4 Neurosurgery, Redlands Community Hospital, Redlands, USA; 5 Neurosurgery, Desert Regional Medical Center, Palm Springs, USA; 6 Neurosurgery, Arrowhead Regional Medical Center, Colton, USA

**Keywords:** neuroendovascular simulator, nir

## Abstract

Simulators for surgical procedures and interventions have undergone significant technological advancement in the past decade and are becoming more commonplace in medical training. Neurosurgery residents across multiple training levels underwent performance evaluation using a neuro-interventional simulator, employing a variety of metrics for assessment. We identified seven core metrics used in the evaluation of neurosurgery residents performing simulated mechanical thrombectomies. Additionally, a systematic PubMed search for studies related to Neurointerventional Radiology training via simulation was performed. The purpose of this study is to examine the validity and benefits of training with these simulation devices and compare our institution's experience. Additionally, an exploration of their applicability to neurosurgery resident training is discussed.

## Introduction

Neurointerventional Radiology (NIR) has evolved from diagnosing diseases (by performing cerebral angiograms) to treating multiple intracranial pathologies in a minimally invasive manner. This is primarily due to advancements in endovascular technologies which has allowed us to treat an array of pathologies such as vascular malformations, aneurysms, ischemic strokes, and tumors. Novel procedures continue to appear yearly, and the current medical practitioner is challenged to keep pace with the developments of the field. With such variety comes an increased onus on training, and various biomedical companies have emerged as industry partners to meet this demand. Companies offering simulators include Mentice (Gothenburg, Sweden) and Simbionix (Airport City, Israel), manufacturers of the vascular intervention simulation trainer (VIST) and ANGIO Mentor, respectively [1]. These two machines comprise a significant portion of the literature on NIR training.

Our institution employed the Mentice NIR simulator in the training of neurosurgery residents, both as preparation for neuroendovascular procedures and as a refresher for more senior residents. We hypothesized that progression in training over time improves performance; senior residents are generally expected to develop and improve upon their excellent medical knowledge when compared to junior residents. We further hypothesized that lower post-graduate year (PGY) levels were more likely to have handling errors, less of an appreciation for dissecting arteries, as well as less judicious in the use of contrast and fluoroscopic exposure to patients.

The purpose of this study was to examine the validity and benefits of training with these simulation devices and report our institution's experience. Moreover, an exploration of their applicability to neurosurgery resident training is discussed. Lastly, a systematic PubMed search for studies related to the use of simulators for Neurointerventional training was performed.

## Materials and methods

**Figure 1:**
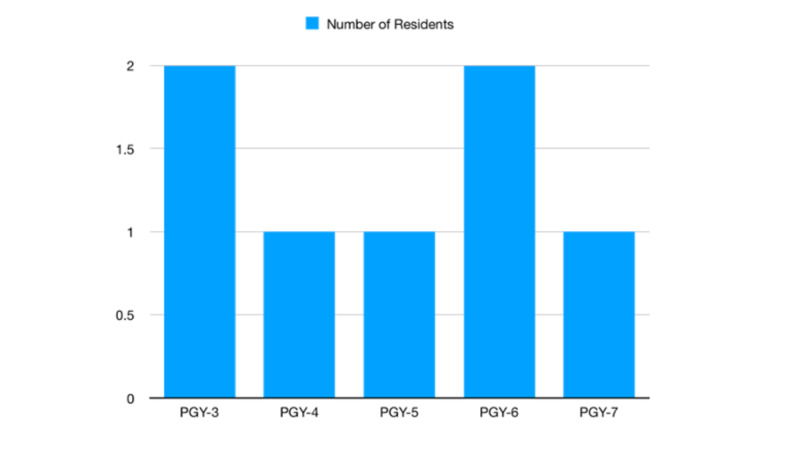
Distribution of program year training among neurosurgery residents performing simulated procedure.

Our study involved the evaluation of performance through multiple metrics across multiple levels of medical education. A standard neurosurgery residency comprises seven years with included exposure to NIR. Neurosurgery residents at the Riverside University Health System Residency program (Moreno Valley, CA) were recruited to perform variations on a simulated mechanical thrombectomy in the left M1 segment of the middle cerebral artery (MCA). Distribution of participants by program year is shown in (Figure 1). Experience per PGY level is demonstrated in Table 1.

**Table 1:** Amount of experience with cerebral angiograms by year. PGY- post-graduate year

Participant	1	2	3	4	5	6	7
PGY	3	3	4	5	6	6	7
Experience	Limited	Limited	Extensive	Extensive	Extensive	Moderate	Moderate

**Figure 2:**
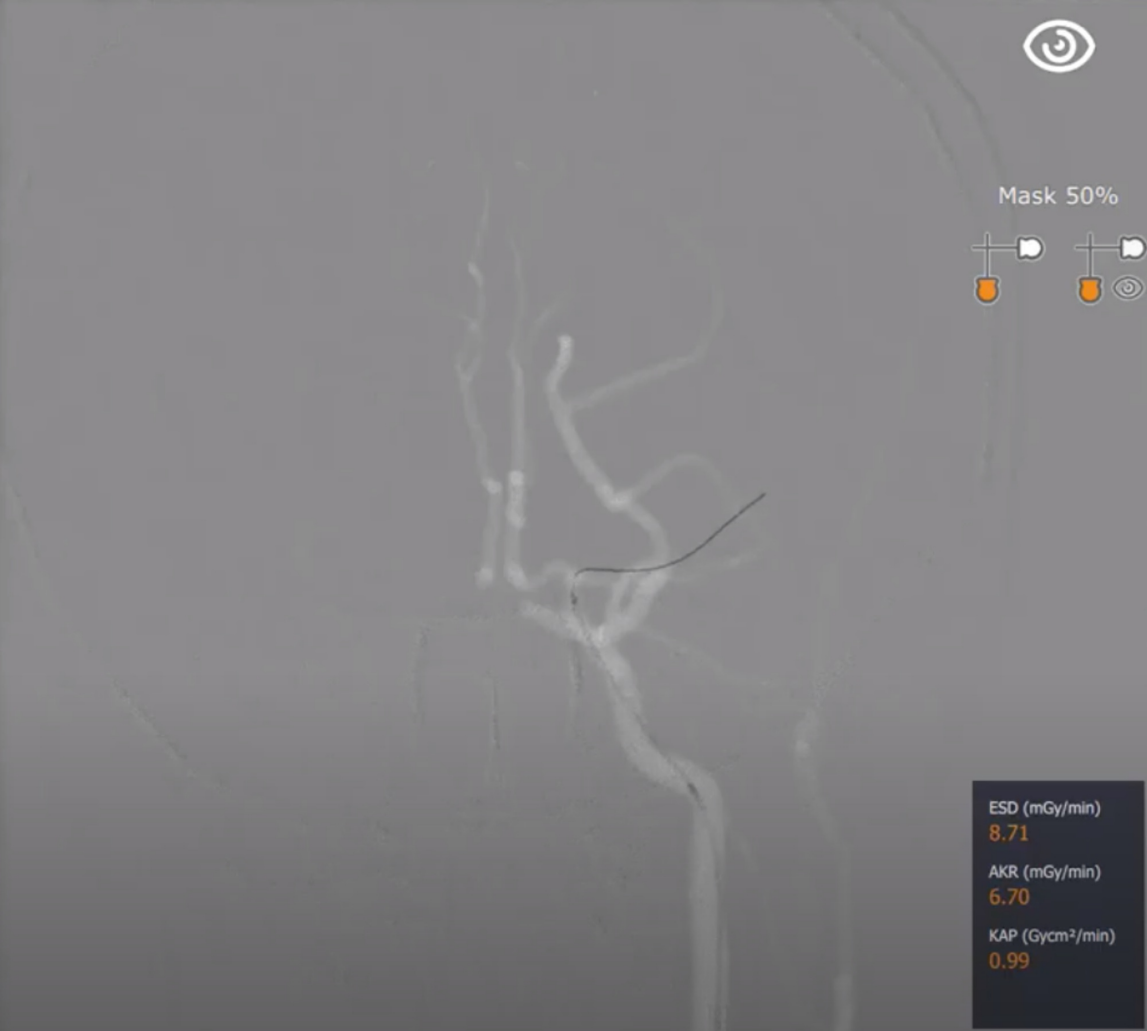
Re-catheterization and positioning of the aspiration catheter.

For our simulated mechanical thrombectomy (Figure 2), the Mentice VIST® G5 simulator (Gothenburg, Sweden) was used, which employs a variety of metrics. For our purposes, and ease of use, we identified seven core metrics used in the evaluation of neurosurgery residents performing simulated mechanical thrombectomies. These included a total time of procedure in seconds, a number of phases finished, steps finished within each phase, a number of handling errors, contrast used in millilitres, total radiation dose, total fluoroscopic time in seconds, and total digital subtraction angiography (DSA) time in seconds. Though the total time was a critical component of the evaluation of the neurosurgery resident's performance during these simulations, better times may be achieved at the subsequent expense of more handling errors, more contrast used, and longer fluoroscopic times. We decided to employ a 2x penalty on handling errors in order to emphasize the importance of surgical skill and hand-eye coordination. Except for the phases/steps completed, all other metrics were evaluated by their resultant initial values. Not all steps were recorded by the system as completed, though all performances were supervised by an attending physician who verified completion of the procedure accurately. Therefore, phases or steps completed and not recorded by the simulator were excluded secondary to simulator error.

## Results

Among the residents performing the simulated procedure, experience with cerebral angiograms in patients varied from no experience to dozens of prior cerebral angiograms (Table 1). No participants had performed a solo mechanical thrombectomy in the past. A total of seven neurosurgery residents participated in the study. The PGY level of each participant and a summation of their respective number of months spent on a dedicated NIR rotation, subspeciality interest, and a number of total NIR procedures performed during residency training are included in Table 1; the summation is listed as either limited, moderate, or extensive. The initial metrics for a standard mechanical thrombectomy case involving occlusion of the left M1 branch of the MCA [HG3] can be seen in Table 2. Best total times were achieved by a PGY-3 and PGY-4 at 1449 seconds and 1212 seconds, respectively. Though the total time was a critical component of the evaluation of the neurosurgery resident's performance during these simulations, better times may be achieved at the subsequent expense of more handling errors, more contrast used, and longer fluoroscopic times. For this reason, an algorithm (described above) was used in the evaluation of resident performance.

**Table 2:** Initial metrics for a standard mechanical thrombectomy case involving occlusion of the left M1 branch of the middle cerebral artery (MCA). PGY- post-graduate year; DSA- Digital Subtraction Angiography;

First Mechanical Thrombectomy							
Medical Education Level	PGY-3	PGY-3	PGY-4	PGY-5	PGY-6	PGY-6	PGY-7
Total time (s)	1449 s	2397 s	1212 s	790 s	2097 s	2202 s	1934 s
Phases finished	9	6	10	2	10	9	10
Steps finished	31	25	33	5	33	31	33
Number of handling errors	56	147	68	27	126	88	120
Contrast used (mL)	389 mL	352 mL	141 mL	54	733 mL	280 mL	414 mL
Total fluoroscope time (s)	738 s	581 s	480 s	530	857 s	950 s	1031 s
Total DSA time (s)	24 s	7 s	48 s	0	75 s	76 s	126 s

The residents with the best scores from the initial run involving occlusion of the left M1 branch of the MCA further went on to compete in a second run, using a bracket system that narrowed the participants down from seven to four. These results are demonstrated in Table 3. Finally, the best two performers were selected for a head to head match involving a mechanical thrombectomy of a left M1 branch of the middle cerebral artery (MCA) with a Type 2 Aortic Arch. For this round, the PGY-3 was determined to be the superior performer, based on the computation of scoring (1096 s of total time + 114 (57 x 2) handling errors, + 229 mL of contrast used + 489 s of total fluoroscopic time) for a total of 1937 versus the PGY-4's 1940 (958 s of total time + 230 (115 x 2) handling errors + 342 mL of contrast used + 410 s of total fluoroscopic time) as seen in Table 4.

**Table 3:** Initial metrics for a standard mechanical thrombectomy case involving occlusion of the left M1 branch of the middle cerebral artery (MCA). DSA- digital subtraction angiography

Second Mechanical Thrombectomy					
Medical Education Level	PGY-3	PGY-4	PGY 5	PGY-6	PGY-7
Total time (s)	2155 s	1635 s	2097 s	2912	2199
Phases finished	10	10	10	9	8
Steps finished	37	37	33	35	33
Number of handling errors	78	205	126	133	78
Contrast used (mL)	679 mL	322 mL	733.0 ml	387 mL	279 mL
Total fluoroscope time (s)	1459 s	995 s	857 s	2577 s	1055 s
Total DSA time (s)	47 s	38 s	75 s	75 s	56

**Table 4:** Second round metrics for a standard mechanical thrombectomy case involving occlusion of the left M1 branch of the middle cerebral artery (MCA) with a Bovine Arch, comprising the best performance from the initial first round mechanical thrombectomy. DSA- digital subtraction angiography

Third Mechanical Thrombectomy		
Medical Education Level	PGY-3	PGY-4
Total time (s)	1096 s	958 s
Phases finished	10	10
Steps finished	33	33
Number of handling errors	57	115
Contrast used (mL)	229 mL	342 mL
Total fluoroscope time (s)	498 s	410 s
Total DSA time (s)	18 s	41 s

## Discussion

From their inception, prototypes and early models for simulation have been examined for their validity [2]. These devices emerged mainly due to advances in graphics computing power and were predicted to positively impact both resident training and patient care [3]. Recently, in a blinded comparison study, researchers randomized 12 attending interventional cardiologists to either a simulation group or a traditional training group. Following the training period, the physicians of the simulation group demonstrated a significantly lower rate of objectively assessed intraoperative errors. The authors concluded a 17-49% transfer of training from the simulator to the in vivo index case [4]. Further studies involving neurointerventionalists may focus on examining the transfer of training present in NIR simulation training.

Most studies involving interventional radiology (IR) simulators are centered around cardiology procedures. We hope that this study will add to the NIR simulator literature. Several studies have previously evaluated specific metrics utilized in our study. In one study, IR fellows vs Attending level efficiency indices were compared over a year at three intervals (1, 6 & 12 months). For 73 Vascular Intervention Simulation Trainer (VIST) procedures, a proficiency score was calculated as the product of procedure time, fluoroscopy time, tools, and contrast agent volume. Efficiency indices for simulated procedures demonstrated scores comparable to the level of clinical experience [5]. In another study, 24 subjects comprised of 10 beginners (residents) 4 intermediates (cardiologists) and ten experts (cardiologists) each performed five coronary angiographies on the VIST simulator. As in the previous study, metrics, including total procedure time, fluoroscopy time, and contrast volume, were extracted from the simulator and analyzed. The experts outperformed trainees in all metrics measured by the simulator, and the authors concluded that the VIST simulator could distinguish between trainees and experts in coronary angiography [6].

Given the demonstration of validity, studies like these often suggest incorporating these simulations into standardized resident curriculum [7]. Importantly, these recommendations are not confined to any unique type of resident. Such validations have also been demonstrated for the ANGIO Mentor device (Simbionix, Airport City, Israel) for use by vascular surgeons [8]. Moreover, the World Federation of Interventional & Therapeutic Neuroradiology (WFITN) recommend "using simulation for basic training in neuro-intervention and encourage the development of new applications covering all aspects of neuro-interventions" [9]. With the backing of professional medical societies like the WFITN, devices like the VIST are being used in new investigational contexts like crisis management [10] and remote streaming support [11]. 

Simulation-based training is already widespread among multiple residency programs and is not confined to a specific field of medicine. For example, residents of radiology have incorporated VR based simulators to practice fluoroscopy-guided lumbar puncture to reduce patient discomfort [12], and vascular surgery residents have demonstrated reduced procedural time for an endovascular aneurysm repair following training with the ANGIO Mentor [13]. Given the nature of the interventions simulated by the VIST and ANGIO Mentor, it is not surprising that many of the residents utilizing these devices are interventional/surgical in their scope of training. In 2008, a study was published in the Journal of Vascular Surgery that examined two groups using the VIST: the first group was classified as a beginner, and the second was classified as intermediate. All were fourth-year and fifth-year general surgery residents interviewing for vascular fellowship training. After simulation practice, total procedure time, fluoroscopy time, average contrast used, percentage of lesion covered by the stent, placement accuracy, residual stenosis rates, and a number of cine loops utilized were similar between the two groups [14]. The authors noted that the difference between novice and the intermediate experience was not as significant a difference as the difference between novice and expert. In a similar study, novice (three PGY-3 general surgery residents) and expert (One vascular surgery fellow and two attendings physicians) physicians practised using the ANGIO Mentor over 72 cases. Notably, both novice and expert groups demonstrated significant reductions in both mean fluoroscopy time and total procedure time [15].

We hypothesized that progression in training improves performance. Two PGY-3s and two PGY-6s were included in this cohort. There were one each of PGY-4, 5 and 7. We believed it was likely that lower PGY levels were more likely to have handling errors, less of an appreciation for dissecting arteries, as well as less judicious in the use of contrast and fluoroscopic exposure to patients. Notably, the more junior level residents outperformed senior residents in terms of total time, phases/steps completed, number of handling errors, as well as the amount of contrast and fluoroscopic/DSA times. This did not demonstrate a correlation between performance and resident seniority. Previously, we believed that with an increase in medical knowledge (anatomy, procedural steps, complications, etc.) and surgical training, there would be an increase in performance and ability. Instead, our data suggest that the improved performance of the junior residents, specifically PGY3 and PGY4, was correlated to an interest in neurointerventional radiology. The more senior residents involving this study PGY five, six, and seven were interested in the pediatric, skull base, and spine fellowships, respectively. What comparison this has to actual neurointerventional practice is a subject of future studies. Future studies may incorporate attending physicians' performance on neurointerventional simulator in comparison to actual patient outcomes.

Despite the benefits of incorporating such simulation training into an integrated INR curriculum [16], challenges still exist to their dissemination among programs. In addition to the training modules themselves, modalities to capture, edit, present, and distribute the simulator training audio, and visual data remains an added cost to programs [17]. This will require additional staff or time to maximize the benefits of the training modules. Moreover, some researchers claim that personalized data like CT imaging would require "2 to 5 days work by a computer engineer/scientist to produce a simulation of sufficient fidelity that would be acceptable for a proficiency-based progression simulation training curriculum" [18]. Such time and financial constraints are a limiting factor for smaller residency programs that may operate outside the help of major academic medical centres.

Of note, a systematic review of simulation-based training in neurosurgery identified 11 studies under the heading of "Neurointerventional Radiology" [19]. Much like the previous studies involving vascular surgery residents, many studies involving neurosurgery residents illustrate decreased procedure times, fluoroscopy doses, and adverse technical events following training with simulators like the Angio Mentor [20].

Limitations include a relatively small sample size of neurosurgery residents as well as limitation of the device itself. Though a sophisticated simulator, there were errors at times with the device recognizing phases were complete, as well as recognition of catheter and guidewire placement within the device. This will eventually be remedied as the state of the art of neuroendovascular simulation proceeds forward. Future studies may evaluate the possibility of performing patient-specific simulations in preparation for neuroendovascular procedures.

## Conclusions

Advances in technology have enabled a new training paradigm using simulators. Many of these have proven validity and offer some benefits to patients. Trainees can benefit from them regardless of their current place in the medical hierarchy. Notably, the benefits of INR simulation are not confined to any one type of resident. Many studies are implicating a place for these devices among neurosurgery residents. While interest remains high, limitations of evidence and barriers to the widespread use of these simulators remain salient issues.
